# Factors associated with the coexistence of anemia and undernutrition among children aged 6–59 months in Mali, 2023/24: A multilevel mixed-effects analysis

**DOI:** 10.1371/journal.pone.0351864

**Published:** 2026-06-25

**Authors:** Halid Worku Jemil, Sonia Worku Semayneh, Eyob Tilahun Abeje, Altaseb Beyene Kassaw, Gosa Mankelkl, Anmut Endalkachew Bezie, Adisu Meles Kabtyimer

**Affiliations:** 1 Department of Health Informatics, College of Medicine and Health Science, Wollo University, Dessie, Ethiopia; 2 Department of Oncology, Addis Ababa University College of Health Science Tikur Anbessa specialized hospital, Addis Ababa University, Addis Ababa, Ethiopia; 3 Department of Epidemiology and Biostatistics, School of Public Health, College of Medicine and Health Sciences, Wollo University, Dessie, Ethiopia; 4 Department of Biomedical Science, College of Medicine and Health Science, Wollo University, Dessie, Ethiopia; 5 Department of Occupational and Safety, College of Medicine and Health Science, Wollo University, Dessie, Ethiopia; Universidade de Sao Paulo Faculdade de Saude Publica, BRAZIL

## Abstract

**Introduction:**

coexistence of anemia and undernutrition is a major public health concern among children in Mali. However, there is a lack of study looking into the relationship between undernutrition and anemia among children in Mali. Therefore, this study was conducted by using multilevel analysis to identify significant factors associated with the coexistence of anemia and undernutrition among children.

**Methods:**

A cross-sectional design was conducted from Mali Demographic and Health Survey data from 2023/24. STATA 17 was used for data summarization and analysis. The model was evaluated using the intra-class correlation coefficient (ICC), median odds ratio (MOR), likelihood ratio (LR), and deviance. Variables with a p-value less than 0.2 in the bi-variable logistic regression analysis were taken into account for the next multilevel analysis. In the multilevel analysis, significant factors were presented using the Adjusted Odds Ratio (AOR) along with the 95% Confidence Interval (CI).

**Results:**

The prevalence of the coexistence of anemia and undernutrition among children was 26.3% (CI: 25.2%, 27.4%). According to the multilevel logistic regression result, mothers aged 25–34 years (AOR = 1.41; 95% CI: 1.01, 1.96), no education (AOR = 1.51; 95% CI: 1.19, 1.91), primary education (AOR = 1.39; CI: 1.05, 1.84), not covered by health insurance (AOR = 2.18; 95% CI: 1.05, 2.47), rural residents (AOR = 1.46; 95% CI: 1.16, 1.84), and short maternal stature (AOR = 1.83; 95% CI: 1.49, 2.27) were associated with an increased odds of the coexistence of anemia and undernutrition among children. In contrast, tall maternal stature (AOR = 0.29; 95% CI: 0.22, 0.36) and children aged 37–47 months (AOR = 0.70; 95% CI: 0.50, 0.96) were associated with decreasing the odds of the coexistence of anemia and undernutrition among children.

**Conclusion:**

In Mali, the coexistence of anemia and undernutrition contributes to mortality and related complications among children. The finding from this study revealed that children whose mothers were aged 25–34, mothers without formal education, mothers without primary education, mothers whose health was not covered by health insurance, children who lived in rural areas, and maternal short stature were associated with increased odds of the coexistence of anemia and undernutrition among children. In contrast, tall maternal stature and children aged 37–47 were associated with decreasing the odds of the coexistence of anemia and undernutrition among children.

## Introduction

Anemia is a condition in which red blood cell or hemoglobin levels fall below an average [1]. Undernutrition refers to a mild, moderate, or severe nutritional deficiency needed for body growth and development. It encompasses stunting, wasting, and underweight [2,3]. According to the WHO, anemia and undernutrition were indicators for assessing hemoglobin deficiency, and micro/macro-nutritional status in children, respectively [4–6]. Both anemia and undernutrition were caused by insufficient iron as well as nutritional deficiencies and recurrent infections [7–9]. Collectively, anemia and undernutrition result in negative health consequences like poor ability to carry oxygen in the blood [10], poor emotional well-being [11], delays in body growth [12–15], impaired immune systems [16–18], poor cognitive and mental development in children [17,19,20], an increased chance of chronic disease [21], and a higher susceptibility to infections and pathogens [22]. Generally, the combined effects of both anemia and undernutrition affect development, poor growth, and challenges in motor skills [23], and impair physical and social well-being [24,25].

According to the World Health Organization, in 2017, 41.7% of children worldwide under the age of five were affected by anemia [26,27]. In 2019, 89% of all disabilities in poor nations were caused by anemia, with 21%, 18%, and 1% of children suffering from mild, moderate, or severe anemia, respectively [28]. Additionally, the coexistence of anemia and undernutrition was more exacerbated in developing countries [29,30]. Similarly, the coexistence is higher in poor-income countries contribute to more than half of global deaths among children under five years [31]. Likewise, both anemia and undernutrition were significantly higher in Western Africa [29]. In 2018, the Mali Demographic and Health Survey reported that 88% of children under five are considered anemic [32]. According to the Mali Demographic and Health Survey, the prevalence of undernutrition among under-fives was a decreasing trend over time. In 2010, stunting (47%), underweight (13%), and wasting (4%) [33,34]. In 2015/16, stunting decreased to 37%, underweight (12%), and wasting (3%), and anemia declined from 73% in 2004 to 63% in 2010 and remained constant to 2015/16.

Anemia and undernutrition share a common strong bidirectional biological and phonological connections which needs a thorough examination. Although many studies have been conducted in Mali, most of them examined the risk factors of anemia and undernutrition individually. Hence, the relationship between anemia and undernutrition received little attention in most of the literature so far, and there is a few study looking into the relationship between undernutrition and anemia among children in Mali. Earlier research that examined these topics individually may not truly show how they are connected to one another [35]. A strong emphasis should be given to examining the link between anemia and undernutrition [29,30,35]. Therefore, this study was examined to investigate the coexistence of anemia and undernutrition among children in Mali using multilevel mixed-effect logistic regression analysis from the MDHS 2023/24 dataset.

## Methods

### Data source and data collection tools

We employed secondary data from the MDHS, 2023/24. The DHS is a nationally representative survey that provides overall data on different health and demographic indicators. The DHS contains information about the child’s health and nutrition, including vaccination profiles. The data collectors received training about the interviewing techniques, confidentiality, and respondent safety in Mali. In addition, before administering the questions, informed consent was gained from all respondents. DHS uses a standardized and validated questionnaire where hemoglobin levels are collected by measuring the hemoglobin levels using capillary blood samples obtained via finger or heel prick and analyzed on-site using a portable HemoCue Hb analyzer by using standardized procedures. Similarly, the anthropometric measurements were collected by measuring the children’s height and weight by professionals using calibrated measuring boards and digital scales, in alignment with the standards set by international protocols [36].

### Study area and participants

This study was conducted in Mali; geographically, it is located between latitudes 10°N and 25°N and longitudes 12°W and 4°E. Its capital is Bamako. With a land area of roughly 1,240,192 km², Mali is the eighth-largest nation in Africa. The source population includes all children aged 6–59 months who reside in Mali, while the study population consists of those children who are present in Mali during the enumeration period.

### Sampling method and sample size determination

**Fig 1:**
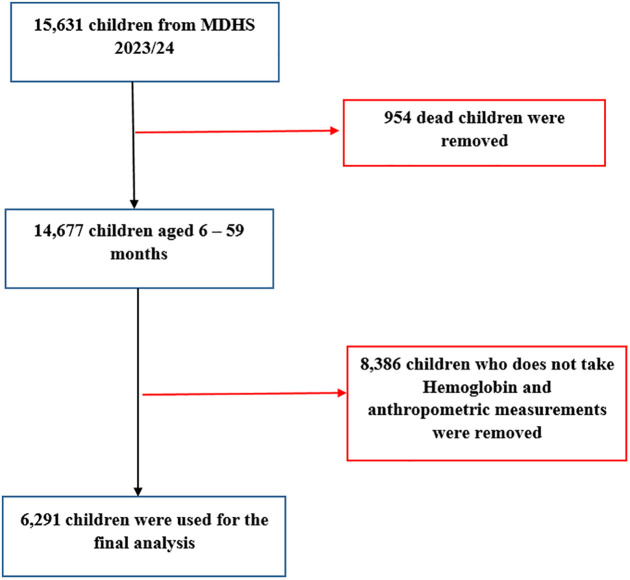
Sample extraction procedure for the coexistence of anemia and undernutrition among children in Mali 2023/24.

The DHS used a cross-sectional design, with a two-stage stratified sampling technique was employed to select representative study participants. In the first stage, enumeration areas were selected using probability proportional to the size of each area, making sure the selection was done independently in every sampling group, stratified by region and urban/rural residence. Then, second, a fixed number of households were systematically chosen from each selected cluster. In DHS the probability of selecting women in households is not uniform; hence, we apply a sampling weight in all analyses to correct for unequal selection and to confirm representativeness. The main DHS indicators were collected from the Measure DHS program website, https://www.dhsprogram.com [37]. A weighted 15,631 children aged 6–59 months were included in this study. From those, 954 dead children and 8,386 children who did not take hemoglobin and anthropometric measurements during the enumeration period were excluded from the study. Finally, a total sample of 6,291 children were used for the final analysis (Fig 1).

### Study variables and operational definition

The outcome variable was the coexistence of anemia and undernutrition (Co-AnUn), operationalized according to the WHO child growth reference standard as the presence of both low hemoglobin levels and inadequate nutritional status.

**Stunting** was dichotomized into a binary category if the child’s Z-score HAZ < −2 SD; the children were categorized as stunted, else not stunted [38–40].

**Wasting** was dichotomized into a binary category; if the child’s WHZ score was less than −2 SD, the children were categorized as wasted, else not wasted [41,42].

**Underweight** was dichotomized into a binary category if the child WAZ-score < −2 SD the children were categorized as underweight, else not underweight [38,43,44].

**Undernutrition** dichotomized into a binary category by merging stunting (if the child’s HAZ is < −2 SD), wasting (WHZ score is < −2 SD), and underweight (WAZ score is < −2 SD). Children were considered undernourished if they met at least one anthropometric failure (HAZ, WHZ, and WAZ Z-scores were < -2 SD). and non-undernourished if their Z-score was >= -2 SD [30,38].

**Anemia** was classified in two binary categories as anemic if the child’s hemoglobin level is < 11 g/dl and not anemic if the hemoglobin level is > 11 g/dl [45–47].

**Coexistence of undernutrition and anemia** they were merged if a child met the criteria for both anemia and at least one of the undernutrition indices (wasting, stunting, or underweight), Anemia ∩ undernutrition indices = Co-AneUn [48,49].

### Independent variables

**Individual-level variables** mother’s occupation, mother’s age, mother’s education, marital status, wealth status, birth order, mother’s height, child age (S1 Fig).

**Community-level variables** place of residence, health insurance coverage, distance to health facility, toilet type, family size, and media exposure (S1 Fig).

**Maternal height** was categorized into three groups: short if a woman’s height is <= 1.50 m, normal if a woman’s height is between 1.5 m and 1.59 m, and tall if a woman’s height is >= 1.60 m [50].

**Media exposure** was dichotomized into two categories by merging three variables together: mothers listening to the radio, watching TV, or reading a magazine. If the mother was exposed to at least one of these, we consider her exposed to media; otherwise, we consider her not exposed to media [51].

### Data management and analysis

To identify the associated factors of Co-AnUn, we first generated descriptive statistics and tabulated the proportion of Co-AnUn and selective independent factors. Similarly, we performed using STATA 17. Similarly, we adjusted the data for both the outcome and predictor variable using DHS sample weights for before analysis and to compute descriptive statics. This helps to correct for unequal probability of selection and to ensure a national representativeness of samples. The multicollinearity was checked before analysis. Further, variables with a p-value < 0.2 in the bivariable analysis were considered in the multilevel analysis.

### Multilevel binary logistic regression analysis

The DHS used multistage sampling techniques from many levels of hierarchy. These methods naturally introduce a correlation among observations within the same cluster. This may give a biased parameter estimate and violate the independence assumption in traditional regression models. To account for this bias from individual and community-level variation, we applied a multilevel binary logistic regression model. We computed the intra-class correlation coefficient (ICC) to quantify the community-level variation. In addition, the Proportional Change in Variance (PCV) was calculated to evaluate the contribution of independent variables in explaining variability across clusters: PCV = [(VA − VB)/VA]100 Where VA = variance of the initial model and VB = variance of the model with more terms. Furthermore, we fitted the following four models (null model, Model II, Model III, and Model IV) sequentially. Model I (Null model): without explanatory variables, to assess baseline variation of Co-AnUn between clusters. Model II: We fit the model by adjusting for individual-level variables (mother’s occupation, mother’s age, mother’s education, marital status, wealth status, birth order, mother’s height, and child age). Model III: We fit the model for community-level variables (place of residence, covered by health insurance, distance to health facility, toilet type, family size, and media exposure) and Model IV (final model): We adjusted for both individual and community-level factors simultaneously. We applied deviance (−2 log likelihood) for model comparison, with the lowest deviance indicating a better model fit. Finally, variables’ with Adjusted Odds Ratios (AORs) with corresponding 95% Confidence Intervals (CIs) in the final model were used. The DHS used a multistage sampling method from many levels of hierarchy that results in a dependency among observations, so to avoid a bias from applying single-level statistical models, we used multilevel modeling techniques.

### Ethical approval

The MDHS were presented without personal identifiers, which is publicly available and obtained from the Demographic and Health Surveys (DHS) Program website http://www.dhsprogram.com through online request. Furthermore, Furthermore, written conscent letter was obtained by the DHS Program Institutional Review Board (IRB).

## Result

### Sociodemographic characteristics of study participants

From 15,631 children, about 6.1% of children were dead and 53.1% of the children did not take hemoglobin and anthropometric measurements during the enumeration period (Fig 1). Most of the participants (mothers/caregivers) (46.19%) were aged 25–34 years, and 24.88% of them were aged 35–49 years. About 62.88% of women had not attained education, 20.49% had secondary education, and 16.63% had primary education. The majority of the participants (77.22%) live in rural areas, and 94% of the participants were not covered by health insurance. The majority of the children (58.70%) were aged 24–36. In addition, nearly half (52.31%) of the participants were not currently working. Regarding media exposure, 78.13% of participants had access to media while 21.87% did not. With respect to marital status, 95.26% were married, 1.61% were not married, and 2.13% were separated (Table 1).

**Table 1:** Sociodemographic characteristics of the study participants, Mali 2023/24, (N = 6,291).

Variables	Weighted frequency	Percent (%)
*Mothers Age*		
*15-24*	1,820	28.93
*25-34*	2,906	46.19
*35-49*	1,565	24.88
*Mothers occupation*		
*Not-Working*	3,291	52.31
*Working*	3,000	47.69
*Mothers education*		
*No-education*	3,956	62.88
*Primary*	1,046	16.63
*Secondary and Higher*	1,289	20.49
*Marital status*		
*Never married*	101	1.61
*Married*	6,056	95.26
*Divorced/separated*	134	2.13
*Wealth status*		
*Poor*	2,577	40.96
*Middle*	1,540	24.48
*Rich*	2,174	34.56
*Birth order*		
*First*	1,142	18.15
*2-4*	2,978	47.34
*>=5*	2,171	34.51
*Media exposure*		
*Yes*	4,915	78.13
*No*	1,376	21.87
*Mothers height*		
*Normal*	3,542	56.3
*Tall*	1,842	29.28
*Short*	907	14.42
*Child age in months*		
*<24*	1,495	23.76
*24-36*	3,693	58.70
*37-47*	1,061	16.87
*48-59*	42	0.67
*Place of residence*		
*Rural*	4,858	77.22
*Urban*	1,433	22.78
*Distance to health facility*		
*Big problem*	2,283	36.29
*Not a big problem*	4,008	63.71
*Toilet types*		
*Not improved*	2,156	34.27
*Improved*	4,135	65.73
*Family size*		
*1-4*	465	7.39
*5-8*	1,885	29.96
*>9*	3,941	62.65
*Covered by health insurance*		
*No*	5,948	94.55
*Yes*	343	5.45
*Stunting*		
*No*	4,541	72.18
*Yes*	1,750	27.82
*Wasting*		
*No*	5,866	93.24
*Yes*	425	6.76
*Underweight*		
*No*	5,228	83.1
*Yes*	1,063	16.90
*Anemia*		
*No*	1,719	27.32
*Yes*	4,572	72.68

### Prevalence of undernutrition Indices and anemia

**Fig 2:**
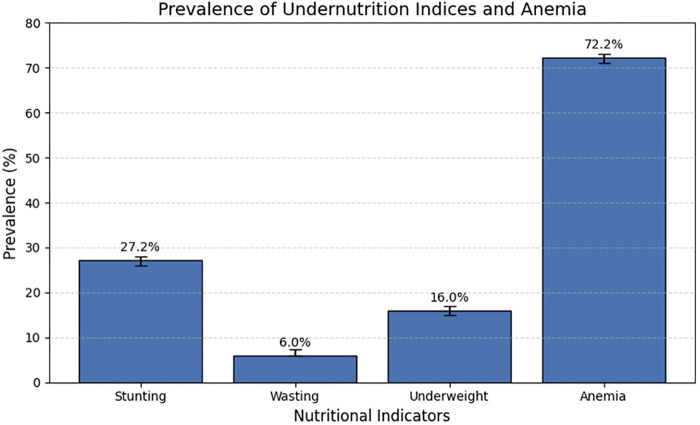
Prevalence of undernutrition Indices and anemia among Children in Mali, 2023/24.

The prevalence of stunting, wasting, underweight, and anemia was 27.2% (95% CI: 26% − 28%), 6% (95% CI: 6% − 7.4%), 16% (95% CI: 15% − 17%), and 72.2% (95% CI: 71% − 73%), respectively. The overall prevalence of Co-AnUn was 26.3% (CI: 25.2%, 27.4%) (Fig 2).

### Random effect analysis

The results of the random-effects analysis in Table 2, the null model (M0), which contained no predictor variables, the between-cluster variance was 0.33, with an ICC of 9.1%, indicating modest clustering in the co-occurrence of anemia and undernutrition among children aged 6–59 months. The median odds ratio (MOR) was 1.73, suggesting considerable heterogeneity across clusters. When individual-level covariates were included in Model 1 (M1), the cluster variance slightly decreased to 0.19, with an ICC of 5.6% and an MOR of 1.51. This model showed a deviance reduction to 5722 compared with the null model. Model 2 (M2), which included only contextual-level variables, showed a reduction in community variance to 0.22, corresponding to an ICC of 6.3% and an MOR of 1.56. The PCV was 33.3%, indicating that contextual factors explained a notable portion of the variance between clusters.

**Table 2:** Random effect analysis for coexistence of anemia and undernutrition among under five children in Mali, 2023/24.

*Model*	*Variance*	*ICC(%)*	*MOR*	*Deviance*	*PCV(%)*
*M0(Null)*	0.33	9.1	1.73	5952	
*M1(Individual)*	0.19	5.6	1.51	5722	42.4
*M2(Community)*	0.22	6.3	1.56	5874	33.3
*M3(Full)*	0.17	4.9	1.48	5697	48.4

**Hint:** *ICC; Inter cluster correlation coefficient, MOR; Median odds ratio, AOR; Adjusted odds ratio, PCV; Proportional change in variance.*

In the full model (M3), which incorporated both individual and contextual-level variables, the community-level variance was further reduced to 0.17. The ICC decreased to 4.9% with an MOR of 1.48, while the deviance was minimized (5697), suggesting the best model fit. The PCV in the full model was 48.4%, indicating that the combined effect of individual and contextual variables explained part of the between-cluster variance. Overall, the random-effects estimates demonstrate significant clustering of Co-AnUn at the community level. The contextual model explained the largest proportion of variance reduction, while the full model provided the best overall fit (Table 2).

### The fixed effect analysis result

In the final multilevel mixed-effects logistic regression model, mothers aged 25–34 years, mothers living in rural areas, mothers with lower educational attainment, mothers without health insurance coverage, and mothers with short stature were significantly associated with an increased likelihood of Co-AnUn among children, whereas tall maternal stature and children aged between 37 and 47 months were associated with a lower likelihood of Co-AnUn among children (Table 3).

**Table 3:** multi-level analysis of Factors associated with coexistence of anemia and undernutrition among under five children in Mali, 2023/24.

Variables and their category	Model 0 (null) AOR 95%CI	Model I AOR 95%CI	Model II AOR 95%CI	Model III AOR 95%CI
*Mothers Age*				
*15-24*		1.43 (0.90, 2.28)		1.43 (0.89, 2.29)
*25-34*		1.39 (1.00, 1.93)**		1.41 (1.01, 1.96)**
*35-49*		1		1
*Mothers occupation*				
*Not-Working*		1.11 (0.95,1.30)		1.10 (0.94, 1.29)
*Working*		1		1
*Mothers education*				
*No-education*		1.61 (1.28, 2.02)**		1.51 (1.19, 1.91)**
*Primary*		1.45 (1.10,1.91)**		1.39 (1.05, 1.84)**
*Secondary and Higher*		1		1
*Marital status*				
*Never married*		1.13 (0.54, 2.37)		1.37 (0.54, 2.37)
*Married*		1.36 (0.76, 2.46)		1.13 (0.76, 2.48)
*Divorced/separated*		1		1
*Wealth status*				
*Poor*		1.49 (1.21, 1.83)		1.13 (0.87, 1.46)
*Middle*		1.41 (1.13, 1.77)		1.15 (0.89, 1.48)
*Rich*		1		1
*Birth order*				
*First*		0.85 (0.63, 1.15)		0.89 (0.66, 1.20)
*2-4*		1.01 (0.71, 1.04)		0.88 (0.73, 1.07)
*>=5*		1		1
*Media exposure*				
*Have Media Exposure*		1		1
*No Media Exposure*		0.75 (0.52, 1.09)		0.76 (0.53,1.10)
*Mothers height*				
*Normal*		1		1
*Tall*		0.29 (0.22, 0.36)**		0.55 (0.43, 0.65)**
*Short*		0.55 (0.44, 0.67)**		1.84 (1.49, 2.27)**
*Child age*				
*>24*		1		1
*24-36*		0.81 (0.51, 2.60)		0.83 (0.66, 1.03)
*37-47*		0.68 (0.49, 0.94)**		0.70 (0.50, 0.96)**
*48-59*		0.70 (0.30, 1.66)		0.73 (0.31, 1.71)
*Place of residence*				
*Rural*			1.82(1.50, 2.21) **	1.46 (1.16, 1.84)**
*Urban*			1	1
*Distance to health facility*				
*Big problem*			1.11(0.95, 1.30)	1.05 (0.90, 1.24)
*Not a big problem*			1	1
*Toilet types*				
*Not improved*			1.19(0.97, 1.46)	1.08 (0.87,1.34)
*Improved*			1	1
*Family size*				
*1-4*			1	1
*5-8*			1.37(0.71, 1.82)	1.17 (0.72, 1.90)
*>9*			1.13(0.92, 2.02)	1.42 (0.95, 2.13)
*Covered by health insurance*				
*No*			2.08(1.34, 3.23)**	2.18 (1.05, 2.47)**
*Yes*			1	1
*Model fit statistics*				
*AIC*	5956.129	5757.296	5890.696	5743.151
*BIC*	5969.317	5875.988	5943.448	5901.406

Hint: *P-value < 0.05, **P-value < 0.001, CI; Confidence interval, AIC – Akaike Information Criterion, BIC-Bayesian Information Criterion.

## Discussion

The overall prevalence of Co-AnUn among children in Mali was 26.3% (CI: 25.2%, 27.4%). We used the univariate, bivariate, and multilevel logistic regression models to assess the associated factors of coexistence of anemia with undernutrition among children. In the multilevel mixed-effect logistic regression model, mothers aged 25–34 years, rural residence, lower maternal educational attainment, lack of health insurance coverage, and short maternal stature were significantly associated with a higher likelihood of Co-AnUn among children. In contrast, tall maternal stature and children aged 37–44 months were associated with a lower likelihood of Co-AnUn among children.

As a result, children whose mothers aged between 25 and 34 years had 1.41 times higher odds of Co-AnUn among children compared to older mothers (AOR = 1.41; 95% CI: 1.01, 1.96). This finding is consistent with a cross-sectional study conducted in Ghana (2019), which reported that children whose mothers were aged below 20 years were 9 times more likely to be underweight and anemic (AOR = 9.455, p = 0.017) compared to mothers who were aged above 40 years [52]. This might be due to infants born from younger mothers being more likely to be born preterm and with a low birth weight, making them more vulnerable to infections and malnutrition [53,54]. This condition may increase the risks of developing anemia, malnutrition, and neonatal infections; these could further increase the risk of developing both anemia and undernutrition [55].

Additionally, children born from mothers without formal education had 1.51 times higher odds of having Co-AnUn compared to mothers with secondary and higher education (AOR = 1.51; 95% CI: 1.19, 1.91). These findings were similar to a study’s findings in Ethiopia from EDHS (2016), which reports that children whose mothers did not have formal education had 1.22 times higher odds of having Co-AnUn compared to mothers with secondary and higher education (AOR = 1.22, 95% CI: 0.73, 0.92) [55]. Similarly, a cross-sectional study conducted in Bangladesh (2016) reported that mothers without formal education had 1.98 times higher risk of having the coexistence of stunting, wasting, and underweight compared to mothers with secondary and higher education (RR = 1.98; 95% CI: 1.25–3.15) [31]. This might be explained by maternal education being essential for increasing mothers’ awareness of their infants’ health and nutrition, including topics such as exclusive breastfeeding and appropriate complementary feeding practices. Understanding proper feeding practices leads to a higher quality of health for a child [56], additionally, mothers’ educational backgrounds can have a positive impact on the health care and nutritional practices they apply to their children [57]. Furthermore, it is obvious that educated mothers have more control over allocating resources for their children’s well-being. This can also be linked to the better parenting procedures practiced by educated women compared to those who lack education [54].

In addition, children whose mothers’ health was not covered by insurance had 2.18 times higher odds of having Co-AnUn compared to mothers whose health was covered by insurance (AOR = 2.18; 95% CI: 1.05, 2.47). A cross-sectional study done in SSA found that mothers’ health insurance coverage decreases childhood stunting and underweight. Similarly, a systematic review and meta-analysis study in Ghana, report that health insurance was found to be a protective factor against anemia among children [58]. This might be due to inequalities in health care financing and lower access, despite equity being one of the key factors in health care systems. There is evidence that the impoverished experience more severe repercussions from morbidity and mortality and have less access to health care than the wealthy. Despite having greater health requirements and spending a larger percentage of their income on medical care, the poor use health care at lower rates than the wealthy [59]. Equity in health signifies that, in an ideal scenario, each individual should have an equal chance to prioritize their health in order to reach their complete health potential, and no one should be hindered from realizing this potential if it is preventable [60]. Furthermore, sustainability in health care finance refers to the relationship between generating revenue, pooling risks for financial security, and efficiently obtaining services to meet the requirements of all. To achieve sustainability, the three interrelated functions of the health financing system must be fulfilled: revenue collection, risk sharing, and health care service procurement [61].

Besides, children who lived in rural areas were 1.46 times higher odds of having Co-AnUn compared to children who lived in urban counters (AOR = 1.46; 95% CI: 1.16, 1.84).There was similar findings with a cross sectional study in Ethiopia from EDHS 2016 reports that Children who lived in rural areas were 1.41 times higher odds of having coexistence of anemia and stunting compared to children who lived in urban counters AOR: 1.41, 95% CI: (1.10, 1.82) [62]. Additionally, there were similar findings with a cross-sectional study in Ethiopia from EDHS 2016 reports: children who lived in rural areas had 1.28 times higher odds of coexistence of anemia and undernutrition compared to children who lived in urban areas (AOR = 1.28, 95% CI: 1.05, 1.57) [55]. This is because people living in rural areas are frequently at a disadvantage in terms of living conditions, economic position, and access to essential care services like vaccination and ANC care [63]. In urban areas, children are less likely to suffer from stunted growth, be underweight, or have any type of undernutrition if their mothers handle home tasks and their fathers work or operate their own businesses. On the other hand, children who have working mothers and fathers in business or service occupations are more likely to be underweight in metropolitan regions. This might be explained by the difficulty working women have finding enough time to provide a balanced meal for their kids [64].

Likewise, children of mothers with short stature were 1.84 times more likely to have Co-AnUn compared to mothers with normal stature (AOR = 1.83; 95% CI: 1.49–2.27). This finding was similar to a cross-sectional study conducted in Pakistan in 2019, which reported that short maternal stature had 1.90 times more likelihood of having coexisting forms of malnutrition compared to mothers with average stature (1.90 (1.02–3.51)) [65], There were findings similar to a cross-sectional study in Ethiopia from EDHS 2016, which reported that mothers with short stature were 2.04 times more likely to have the coexistence of anemia and stunting compared to mothers with tall stature [62], because of this, the association may be explained by an intergenerational cycle of malnutrition in which stunted women children grow up to become stunted mothers, who then give birth to stunted children [66]. Furthermore, due to the intergenerational cycle of stunting, underweight mothers are more likely to produce stunted children, and stunted children are more likely to be anemic. Hemoglobin (Hb) levels and HAZ were found to be positively correlated [40,67].

In contrast, tall maternal stature was 71% less likely to have Co-AnUn compared to mothers with average stature (AOR = 0.29; 95% CI: 0.22, 0.36). This finding is consistent with a cross-sectional study conducted in Pakistan in 2019, which reports that tall maternal stature had 47% fewer times the likelihood of having Co-AnUn compared to mothers with average stature (AOR = 0.53; 95% CI: 0.28–0.98) [65]. Similarly, a cross-sectional study done in 54 LMICs [68], reports that a 1 cm increase in maternal height was associated with a 1.2% reduction in underweight (RR = 0.968; 95% CI: 0.968–0.969) and a 3.2% reduction in stunting (RR = 0.968; 95% CI: 0.967–0.968) [68]. This might be explained by shorter maternal height tending to possess narrower pelvic structures, which raises the chances of cephalopelvic disproportion and difficulties during childbirth, resulting in obstructed labor [69]. Similarly, in shorter mothers who might possess reduced health reserves, the delivery of essential nutrients to the fetus may prove insufficient, causing intrauterine growth restriction and diminished birth weight, factors that can affect the health and survival of the offspring. For these mothers, a restricted supply of nutrients at the cellular level throughout their development may result in the prioritization of fundamental metabolic functions, thereby causing resources to be redirected from growth, which ultimately leads to retarded growth and reduced height [70].

Lastly, children aged 37–47 months were 30% less likely to have Co-AnUn compared to children with short stature (AOR = 0.70; 95% CI: 0.50–0.96). It is consistent with a cross-sectional study reported in Ethiopia, 2014, with lower odds of stunting among children aged 36–47 months (AOR  =  0.41; 95% CI: 0.22, 0.78) [71], similarly, a cross-sectional study in Ethiopia in 2016 reported 25% lower odds of anemia among children aged 36–47 months (AOR = 0.25, 95% CI: 0.20, 0.31) [72]. This could be explained by the finding that children between the ages of 37 and 47 months had less capacity to store nutrients than children older than 37 months. Children who have a low capacity for nutritional reserves and consistently consume low-quality supplemental meals are more likely to experience negative developmental outcomes. Early childhood is one of the most crucial stages in terms of the possibility of stunted growth and its long-term effects, according to additional studies [73].

### Strengths and limitations

This study used standardized measurements from WHO and DHS guidelines for measuring undernutrition indices such as stunting, wasting, underweight, and anemia to determine Co-AnUn, which are internationally recognized and widely used by the WHO and other organizations as a standardized tool for measuring undernutrition indices. Subsequently, comparison of results across many studies will be possible. Additionally, the hierarchical nature of DHS data is conducted using an advanced model to take into account the clustering effect (mixed-effect logistic regression) for a reliable standard error and estimate.

However, the study design was cross-sectional; it does not show a causal relationship or long-term effects. The secondary nature of the dataset makes it difficult to obtain and analyze additional variables due to the absence of micronutrient intake data in the DHS dataset. Furthermore, DHS lacks dietary covariates, preventing analysis of micronutrient deficiencies, including zinc and vitamins, which are known drivers of Co-AnUn. The absence of nutritional covariates precludes any analysis of micronutrient deficiencies. Therefore, we recommend future research be conducted using longitudinal designs to track causal relationships and incorporation of dietary assessment tools.

## Conclusion

The coexistence of anemia with undernutrition is still prevalent in Mali, which contributes to mortality and related complications among children. The finding from this study revealed that children whose mothers were aged 25–34, mothers without formal education, mothers without primary education, mothers whose health was not covered by health insurance, children who lived in rural areas, and maternal short stature were associated with increased odds of the coexistence of anemia and undernutrition among children. In contrast, tall maternal stature and children aged 37–47 were associated with decreasing the odds of the coexistence of anemia and undernutrition among children.

### Implications of the study

The findings of this study carry significant implications for public health policy and nutritional intervention strategies in Mali. Improving equity of quality health services for limited healthcare access populations. Programs should prioritize the first 1,000 days of infants and newborn care by improving maternal education. Furthermore, the study highlights the critical importance for healthcare systems to integrate nutritional supplements with maternal and child health services, particularly targeting disadvantaged populations through community-based outreach programs for rural residents and providing insured maternal care services. Educational initiatives should focus on improving health literacy for mothers, while economic policies should address healthcare accessibility to remote areas.

## Supporting information

S1 Fig Conceptual framework for the Coexistence of Anemia and Undernutrition among children in Mali 2023/24 adapted from WHO 2017 and UNICEF 2013 framework. (TIF)

S1 Data STATA dataset. (DTA)

## Abbreviations

AIC: Akaike Information Criterion
CI: Confidence Interval
Co-AnUn: Coexistence of Anemia and Undernutrition
DHS: Demographic and Health Survey
HAZ: Height for Age
MDHS: Mali Demographic and Health Survey
WAZ: Weight for Age
WHZ: Weight for Height
WHO: World Health Organization
