# Supplementary figures and images for: Factors associated with the coexistence of anemia and undernutrition among children aged 6–59 months in Mali, 2023/24: A multilevel mixed-effects analysis
Source: PLoS One. 2026 Jun 25;21(6):e0351864. doi: 10.1371/journal.pone.0351864 (PMC13298962; doi:10.1371/journal.pone.0351864)

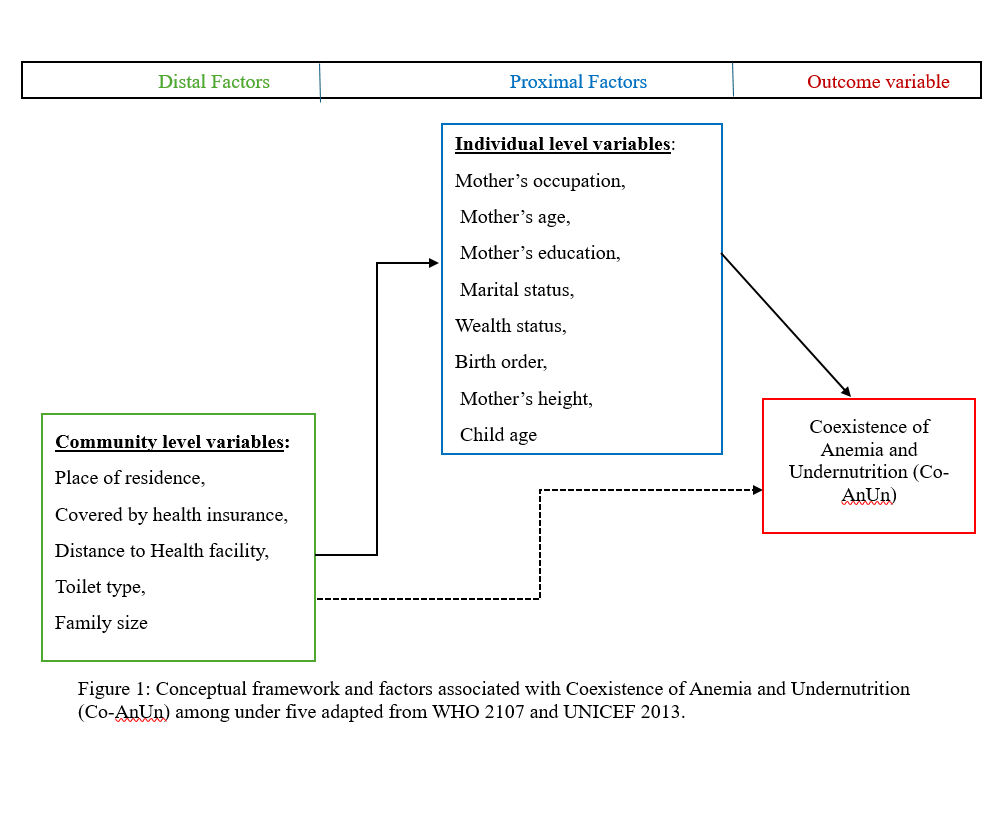

Supplement: S1 Fig — (TIF) [file pone.0351864.s001.tif]
